# Experiences of menstrual inequity and menstrual health among women and people who menstruate in the Barcelona area (Spain): a qualitative study

**DOI:** 10.1186/s12978-022-01354-5

**Published:** 2022-02-19

**Authors:** Anna Sofie Holst, Constanza Jacques-Aviñó, Anna Berenguera, Diana Pinzón-Sanabria, Carme Valls-Llobet, Jordina Munrós-Feliu, Cristina Martínez-Bueno, Tomàs López-Jiménez, Mª Mercedes Vicente-Hernández, Laura Medina-Perucha

**Affiliations:** 1 Fundació Institut Universitari per a la recerca a l’Atenció Primària de Salut Jordi Gol i Gurina (IDIAPJGol), Gran Via de les Corts Catalanes, 587 attic, 08007 Barcelona, Spain; 2 Universitat Pompeu Fabra, Barcelona, Spain; 3 Universitat Autònoma de Barcelona, Bellaterra (Cerdanyola del Vallès), Spain; 4 Departament d’Infermeria, Universitat de Girona, Girona, Spain; 5 SomiArte Taller, Barcelona, Spain; 6 Centro de Análisis y Programas Sanitarios (CAPS), Barcelona, Spain; 7 Atenció a la Salut Sexual i Reproductiva (ASSIR) Muntanya/La Mina, Institut Català de la Salut, Barcelona, Spain; 8 Servei d’Atenció a la Salut Sexual i Reproductiva (ASSIR). Direcció Assistencial d’Atenció Primària, Institut Català de La Salut, Barcelona, Spain; 9 Sexual and Reproductive Health Care Research Group (GRASSIR), Barcelona, Spain

**Keywords:** Menstrual health, Menstrual inequity, Menstrual equity, Period poverty, Menstrual hygiene management, Androcentrism, Menstruation, Social inequities of health, Salud menstrual, Inequidad menstrual, Equidad menstrual, Pobreza menstrual, Manejo menstrual, Androcentrismo, Menstruación, Inequidades sociales en salud

## Abstract

**Introduction:**

Menstrual health and menstrual inequity have been neglected in social, economic, healthcare and political spheres. Although available evidence is scarce, it already suggests a link between experiencing menstrual inequity (which refers to the systematic disparities in accessing menstrual health and education, menstrual products and spaces for menstrual management, among other aspects) and menstrual health outcomes. The aim of this study was to explore experiences of menstrual health and menstrual inequity among women and people who menstruate aged 18–55 in Barcelona and surrounding areas (Spain).

**Methods:**

A qualitative study, using a critical feminist perspective, was conducted. Sampling was purposeful and selective. Recruitment was through sexual and reproductive health centres, social media and snowball sampling techniques. Thirty-four semi-structured photo-elicitation interviews were conducted between December 2020 and February 2021. Interviews took place in sexual and reproductive health centres, public spaces, and by telephone. Data were analysed using Reflexive Thematic Analysis.

**Results:**

Three themes were identified: “Systemic neglect of menstruation and the menstrual cycle”, “When “the private” becomes public: menstrual management” and “Navigating menstrual health: between medicalization and agency”. Experiences of menstrual inequity appeared to be widespread among participants. They referred to the impact of having to conceal menstruation and the barriers to managing menstruation in public spaces. Choosing menstrual products was often influenced by price and availability; several participants reported menstrual poverty. A general lack of menstrual education was described. Menstrual education was usually gained through personal experience and self-learnings, or through families and friends. Menstruation and the menstrual cycle had a significant impact on participants’ day-to-day. Accessing and navigating the healthcare system was challenging, as participants mostly reported feeling dismissed and almost exclusively offered hormonal contraception as a panacea to address menstrual health.

**Conclusions:**

The impact of menstrual inequity appears to be far-reaching. Multidimensional structural policies should promote agency in individuals and communities to enable opportunities for menstrual education, access to menstrual products, healthcare services and adequate menstrual-management facilities. Health professionals’ training is also necessary to improve access to and quality of menstrual healthcare. Policies need to be inclusive of non-binary and trans people, and vulnerable populations.

**Supplementary Information:**

The online version contains supplementary material available at 10.1186/s12978-022-01354-5.

## Plain language summary

There is a lack of research on menstrual health and menstrual inequity. However, we already know that menstrual inequities may have a negative effect on menstrual health. Yet, politicians, policymakers and healthcare professionals rarely pay attention to menstrual inequity and menstrual health. This study had the objective to collect experiences of menstrual inequity and menstrual health among women and people who menstruate (trans and non-binary people) between 18 and 55 in the Barcelona area (Spain). Thirty-four participants took part in interviews between December 2020 and February 2021. We used photographs during the interviews to deepen discussions with participants. We did the interviews in sexual and reproductive health centres, in public spaces and over the phone. Participants shared a variety of experiences of menstrual inequity and health. For instance, some participants explained that they had experienced financial issues to buy menstrual products. Others, that it was often difficult to find adequate spaces to change menstrual products at schools, workplaces and public spaces. Participants told us that they mostly learned about menstruation through their own experiences and talking to family and friends. They had little information on menstruation. Also, when participants sought medical help they usually found that health professionals usually only offered hormonal contraception to treat menstrual issues. They felt frustrated and dismissed. In general, they thought that menstruation affected their daily lives. Therefore, it is very important to train professionals, continue research and put policies for menstrual inequity and health in place.

**Supplementary Information:**

The online version contains supplementary material available at 10.1186/s12978-022-01354-5.

## Resumen

**Introducción:**

La salud y la inequidad menstrual no han sido bien atendidas en las esferas social, económica, sanitaria y política. Aunque la evidencia disponible es escasa, ya sugiere un vínculo entre experiencias de inequidad menstrual (relativa a las dificultades sistemáticas para el acceso a la salud y educación menstrual, productos menstruales y espacios de manejo menstrual, entre otros aspectos) y la salud menstrual. El objetivo de este estudio fue explorar las experiencias de salud e inequidad menstrual en mujeres y personas que menstrúan (18–55 años) en Barcelona y alrededores (España).

**Métodos:**

Estudio cualitativo desde una perspectiva feminista crítica. El muestreo fue intencionado y selectivo. El reclutamiento se realizó en centros de salud sexual y reproductiva, redes sociales y a través de técnicas de bola de nieve. Se realizaron 34 entrevistas semi-estructuradas utilizando foto-elicitación, entre diciembre de 2020 y febrero de 2021. Las entrevistas se llevaron a cabo en centros de salud sexual y reproductiva, espacios públicos y por teléfono. Los datos se analizaron mediante Análisis Temático Reflexivo.

**Resultados:**

Se identificaron tres categorías: “Descuido sistemático de la menstruation y el cycle menstrual”, “Candy “lo privacy” se vuelve public: manejo menstrual” y “Navegando la salad menstrual: entree la medicalización y la agencia”. Las participantes compartieron diversas experiencias de inequidad menstrual. Diversas participantes compartieron experiencias de pobreza menstrual, al no poder acceder o escoger productos menstruales debido a su precio y disponibilidad. Según las participantes, los aprendizajes menstruales son escasos. Estos suelen darse a través de la experiencia propia y el autoaprendizaje, o de familiares y amigas. La menstruación y el ciclo menstrual parecen tener un impacto significativo en el día a día, incluyendo el impacto de tener que ocultar la menstruación y las barreras para manejar la menstruación en los espacios públicos. Se identificaron barreras para acceder y utilizar el sistema sanitario. Las participantes también reportaron la prescripción sistemática de anticonceptivos hormonales como panacea para abordar la salud menstrual.

**Conclusiones:**

Es necesario desarrollar e implementar políticas estructurales y multidimensionales para promover la agencia de las mujeres y personas que menstrúan, así como de las comunidades, para generar oportunidades para aprender sobre la menstruación, acceder a productos menstruales y servicios de atención sanitaria, así como a espacios adecuados para el manejo menstrual. Fomentar la formación de profesionales de la salud es también crucial para mejorar el acceso y la calidad de la servicios para la salud menstrual. Estas políticas deben incluir a las personas trans y no binarias, así como a poblaciones más vulnerables.

## Introduction

Menstruation is a healthy biological event that has recently been considered a vital health sign [1, 2]. *Menstrual health* (MH) can be defined as having menstrual cycles lasting 21–35 days, a menstrual cycle variation of 5–7 days yearly, menstrual flow during 2–7 days and bleeding between 25–80 ml per menstruation [3, 4]. Other authors also indicate that a healthy menstrual cycle should be ovulatory [5]. Recently, MH has been defined from a more holistic perspective, as: “a state of complete physical, mental, and social well-being and not merely the absence of disease or infirmity, in relation to the menstrual cycle.” [6]*.* However, MH is often disregarded in healthcare and not included in public health strategies [7], despite experiencing MH issues (e.g., dysmenorrhea) could greatly compromise quality of life [8]. This structural neglect is explained by how ingrained androcentric values and norms (i.e., considering male’s needs, priorities, values and stereotypical physical characteristics as “the gender-neutral standard”) are in social, political and research systems [9, 10]. Based on this, menstruation cannot be considered normative within the lens of androcentrism [10].

Based on the current study, the authors have conceptualized *menstrual inequity* (MI) as a two-fold concept (Medina-Perucha et al., *in prep*). First, MI can be understood in comparison to men and other non-menstruating people (out-group MI), as “the systematic and avoidable disparities in the access to healthcare, education and knowledge, experiences of stigma and discrimination, the lack of research on the menstrual cycle and menstruation, and the barriers to social, community, political and economic participation based on having a menstrual cycle and menstruating”. Second, we may consider MI within women and people who menstruate (PWM) (in-group MI) as “the systematic and avoidable disparities between different populations in the access to menstrual healthcare, menstrual education and knowledge, products, services and facilities for menstrual management, menstrual-related experiences of stigma and discrimination and barriers to social, community, political and economic participation” (Medina-Perucha et al., in prep). This conceptualization of MI encompasses *period poverty* (or menstrual poverty) [11, 12], which refers to financial and material barriers to accessing menstrual products. It also includes *menstrual hygiene management* (or menstrual management), which focuses on the need to ensure safe menstrual management facilities (e.g., spaces that are clean, where water is available and include a bin and can be locked) [11, 13–18]. MI is argued to be the result of androcentric social, economic, political and research systems. Besides, while the link between MI and MH has yet not been established, we argue that addressing MI may have a positive impact on MH outcomes.

Barriers to address menstruation as a natural event for half of the population leads to experiences of stigma and shame, and compromises community participation, equity and free agency [19, 20]. Stigma, shame, taboo and misconceptions around the menstrual cycle and menstruation are prevalent in many cultures [14]. Menstruation is surrounded by negativity, mystery, and ideas of dirtiness [21] and embarrassment [31]. One of the reasons for menstrual concealment and some taboos to prevail is because menstrual education is usually through families [13, 21, 22]. Even if community-based education should be promoted, the population should have better access to accurate menstruation-related information and knowledge. University curricula on healthcare, public health and education professions should also incorporate MH and MI as public health issues [18, 23].

Another consequence of prevailing MIs is the medicalization of the menstrual cycle and menstruation [24, 25]. Hormonal contraception (HC), which generally stops a natural menstrual cycle and menstruation, is a common strategy to manage menstruation-related issues such as menstrual pain or even acne [20, 26]. Women and PWM experiencing menstrual problems rarely attend healthcare services, partly as some menstrual health problems tend to be normalized and dismissed [22, 27, 28]. Delayed diagnoses (e.g., of endometriosis) and negative experiences are expected when seeking institutionalized healthcare for menstrual-related issues [27].

Previous evidence suggests that dysmenorrhea (painful menstruation) is widespread among women and PWM [29] and may be related to absenteeism [22, 29] and presenteeism in the workplace and schools [30] (e.g., working while feeling unwell). On the other hand, community-based and policy actions are focusing on reducing menstrual products’ price. Their cost has been increasingly questioned, since they are rarely taxed as necessity goods and some women and PWM cannot afford them [31–34]. Despite the need to reduce menstrual products’ costs, partly as a matter of social justice, price reductions may not be enough to ensure affordability [32, 33]. In fact, there have been various social movements aiming at normalizing the conversation around menstruation [35], criticizing the cost of menstrual products and the application of the “pink tax” in various countries [32–34]. The “pink tax” refers to products marketed towards women being more expensive than “male products” (e.g., razors).

These social movements have also encouraged the promotion of health literacy [22, 36], the “ability to interpret, maintain, understand and use health decisions and follow treatment instructions” [27]. “Body literacy” may be a more appropriate concept when discussing menstrual health. We define it as: (1) the ability to understand and observe how health and wellbeing are connected to the body’s natural functions and their environment, (2) the knowledge to engage in meaningful discussions with health professionals and (3) the confidence to participate in decisions regarding one’s health [36, 37]. If women and PWM can understand and make decisions regarding their menstrual cycle and menstruation, they can better monitor and self-manage their health [3].

Although half the population menstruates, MH and MI are largely ignored worldwide in social, economic and political spheres. This has translated into a lack of research and inclusion of MH and MI in public health, healthcare and educational agendas [7]. This is inherently associated with the gender research (or data) gap and androcentric and patriarchal systems [7, 10, 24, 38, 39]. Most research on MH, menstrual poverty and menstrual management has been conducted in the Global South, investigating menstrual health and menstrual inequity in the Global North is also imperative [31, 40]. This study aimed at exploring experiences of menstrual health and menstrual inequity among women and PWM aged 18–55 in the Barcelona Metropolitan area. It is part of a larger mixed-methods project, the “Equity and Menstrual Health in Spain” study.

## Methods

This is an explorative qualitative study, using photo-elicitation techniques [41, 42]. This work takes a critical and feminist perspective [43–45], to critically examine systemic neglects and structural violence embedded in institutions and social structures, and how power relations have an impact on society [45–47]. Taking a feminist perspective means considering social inequities from a gender perspective, working to attain freedom, justice and equity and exposing disparities [43, 48, 49]. Reflexivity, considered one of the pillars for critical qualitative research [50], has been an integral part of the research process. The authors have continuously considered how their own perceptions and experiences have influenced the research questions, data collection and analysis. Quality and rigor have been ensured by following the Guba & Lincoln criteria [51], by ensuring the research’s (1) credibility (i.e., confidence in the truths apparent in the findings), (2) transferability (i.e., findings are applicable to the context where the research has conducted and can inform other contexts), (3) dependability (i.e., findings are consistent and the research could be replicated), and (4) confirmability (i.e., findings are true to participants’ accounts and researchers’ motivations, interests and perspectives have been clearly exposed). Research quality has also been evaluated through the Critical Appraisal Skills Programme (CASP) tool [52] (see Additional file 1). The term PWM will be used to be inclusive of people who menstruate that are trans, intersex or non-binary.

### Participants

Study participants were 34 women and PWM aged 18–47 years old, living in Barcelona or surrounding areas. Having entered menopause or have been 1 year or more without menstruating due to ovarian failure, was the main exclusion criteria. One participant (P2) was interviewed unknowingly that she had recently entered early menopause. Her interview was included in the analysis for ethical reasons, as data were already being collected when researchers knew that she was not menstruating regularly. See Table 1 for participants’ sociodemographic information and Additional file 2 for participants’ self-reported menstrual cycle and menstruation characteristics.

**Table 1:** Participants’ sociodemographic characteristics (N = 34)

ID	Age	Residence (city)	Birthplace	Administrative status	Employment status	Economic issues in the last year	Completed education	Gender	Identifies as trans	Roma community	Interview location
P1	27	Sant Adrià del Besos	Spain	Spanish nationality	No employment/income	Yes, sometimes	Primary education	Woman	No	Yes	ASSIR
P2	40	Barcelona	Spain	Spanish nationality	Works full-time	No	Secondary education	Woman	No	No	ASSIR
P3	23	Barcelona	Spain	Spanish nationality	Maternity leave	Yes, sometimes	Professional education	Woman	No	Yes	ASSIR
P4	24	Barcelona	Spain	Spanish nationality	Works full-time	No	University studies	Woman	No	No	Telephone
P5	25	Barcelona	Spain	Spanish nationality	Works full-time	No	University studies	Woman	No	No	Telephone
P6	29	Barcelona	Spain	Spanish nationality	Self-employed	No	University studies	Not sure	Not sure	No	Telephone
P7	33	Sant Cugat del Vallès	Spain	Spanish nationality	Works full-time	No	University studies	Woman	No	No	Telephone
P8	35	Sant Feliu de Llobregat	Spain	Spanish nationality	Works full-time	No	University studies	Woman	No	No	Telephone
P9	24	Barcelona	Spain	Spanish nationality	Works full-time	Yes, always	University studies	Woman	No	No	Telephone
P10	33	Barcelona	Spain	Spanish nationality	Works full-time	No	University studies	Woman	No	No	Telephone
P11	33	Barcelona	Spain	Spanish nationality	Works full-time	No	University studies	Woman	No	No	Telephone
P12	25	Barcelona	Spain	Spanish nationality	Work full-time; Studies part-time	No	University studies	Woman	No	No	Public space
P13	25	Barcelona	Spain	Spanish nationality	Works full-time	No	University studies	Woman	No	No	Public space
P14	26	Barcelona	Spain	Spanish nationality	Studies full-time	No	University studies	Woman	No	No	Public space
P15	25	Sabadell	Spain	Spanish nationality	Works part-time	No	University studies	Woman	No	No	Telephone
P16	47	Tarragona	Spain	Spanish nationality	Works full-time	No	Professional education	Woman	No	No	Telephone
P17	34	La Torre de Claramunt	Spain	Spanish nationality	Works full-time	No	University education	Woman	No	No	Telephone
P18	23	L´Hospitalet de Llobregat	Spain	Spanish nationality	Medical leave; Studies part-time	Yes, sometimes	Professional education	Woman and non-binary	Not sure	No	Telephone
P19	25	Barcelona	Spain	Spanish nationality	Works part-time; Studies full-time	Yes, sometimes	Secondary education	Woman	No	No	Telephone
P20	20	Barcelona	Spain	Spanish nationality	Studies full-time	Not sure	Secondary education	Woman	No	No	Telephone
P21	35	Badalona	Spain	Spanish nationality	Self-employed; Studies part-time;Unpaid houseworker	No	University studies	Woman	No	No	Telephone
P22	18	Barcelona	Spain	Spanish nationality	Studies full-time; Unpaid houseworker	Yes, sometimes	Secondary education	Woman	No	No	Public space
P23	28	Sabadell	Spain	Spanish nationality	Works full-time	No	University education	Woman	No	No	Telephone
P24	20	Sabadell	Spain	Spanish nationality	Studies full-time	No	Secondary education	Non-binary	Yes	No	Telephone
P25	37	Badalona	Morocco	Permanent residence	Works full-time	No	Professional education	Woman	No	No	Telephone
P26	24	Manresa	Spain	Spanish nationality	Works part-time	Yes, sometimes	Professional education	Woman	No	No	Telephone
P27	35	Sabadell	Columbia	Spanish nationality	Unemployed	Yes, sometimes	University studies	Woman	No	No	Telephone
P28	37	Cardedeu	Spain	Spanish nationality	Works full-time	No	University studies	Woman	No	No	Telephone
P29	23	Barcelona	Argentina	Refugee status	No income	Yes, sometimes	Secondary education	Woman	No	No	ASSIR
P30	22	Barcelona	Spain	Permanent residence	Works full-time	Yes, sometimes	Professional education	Woman	No	No	ASSIR
P31	25	Badalona	Pakistan	Permanent residence	Works full-time	Yes, sometimes	Professional education	Woman	No	No	Public space
P32	29	Terrassa	Spain	Spanish residence	Works full-time	No	University studies	Woman	No	No	Telephone
P33	28	Esplugues del Llobregat	Spain	Spanish nationality	Works full-time	Yes, sometimes	University studies	Woman	No	No	Telephone
P34	38	Sant Adria del Besòs	Brazil	Spanish nationality	Unemployed	Yes, sometimes	Professional education	Woman	No	No	ASSIR

*ASSIR:* sexual and reproductive healthcare centre

### Sampling and recruitment

Sampling was purposive and selective. Participants were recruited through social media (Instagram, Twitter and WhatsApp) and relevant key persons and organizations in the Barcelona area such as public sexual and reproductive health centers (ASSIRs). Discourse diversity was ensured by recruiting participants with different characteristics, such as age, socio-economic context, country of birth, gender and being from the Roma community (a distinctive and widespread cultural group in Spain). Special attention was paid to recruiting women and PWM who had limited access to social media, people living in socio-economic deprived areas, participants from the Roma community and migrant populations. Snowball sampling techniques were also used.

### Data collection

Thirty-four semi-structured photo-elicitation interviews were conducted between December 2020 and February 2021. Individual interviews were considered most appropriate since menstruation is often considered a taboo and could be a sensitive topic [53, 54]. Photo-elicitation techniques were chosen to facilitate conversations around menstruation and elicit participants’ responses and participation [41, 42, 46], and allow to reinforce the themes discussed throughout the interviews [55]. A topic guide was developed by the research team (see Additional file 3). Data on participants’ conceptualizations on menstruation and menstrual health, identified opportunities to improve menstrual health and menstrual equity, perceptions on menstrual products, and perceived impact of COVID-19 on their menstrual cycles and menstruation, will be explored in future publications. Two photographs, chosen by the research team, were used during the interviews (see Additional file 4). Photographs were shown to participants, who were asked to discuss the photographs based on how would they describe them and what did they feel about the images portrayed.

Participants were given the option of doing the interview in person or over the phone, depending on their preferences and COVID-19 public health recommendations. Face-to-face interviews (N = 11) were either conducted in ASSIRs (N = 6) or public spaces (N = 5) that allowed enough intimacy for the interviews while COVID-19 prevention measures could be ensured. The rest were telephone interviews (N = 23). For telephone interviews, photographs were sent by email just at the start of the interview. The interviews were conducted by LMP (N = 11) and ASH (N = 11). Some interviews were conducted by both LMP and ASH (N = 12), as ASH was training as a qualitative researcher.

The interviews lasted between 40 min and 1 h and 25 min. All interviews were audio recorded with participants’ consent. Participants received a debrief form with resources on the menstrual cycle and menstruation, and a 10€ voucher as a token of thanks for participating.

### Data analysis

Reflexive inductive thematic analysis [46, 56] was used for data analyses. First, all interviews were transcribed verbatim, anonymized, and prepared for analysis by ASH and external transcribers. Secondly, a preliminary analysis was carried out by ASH and LMP by reading, reflecting, and discussing the data, to formulate preliminary reflections and themes using data from 12 interviews. These interviews were coded by ASH, and triangulated with LMP and CJA by comparing and discussing codes and potential themes and sub-themes resulting from them. Themes and sub-themes were identified inductively, through deep analysis and reflection, as well as discussions between ASH and LMP [56]. A meeting with the whole research team was organized to have an in-depth discussion on the preliminary findings. A preliminary conceptual framework with themes and sub-themes was developed. The remaining 22 interviews were then coded and analyzed based on the conceptual framework developed by LMP and ASH. While new themes did not emerge on this second round of analysis, themes and sub-themes were re-organized. The final thematic framework was finally developed based on team discussions.

## Results

Data analysis identified three themes: “Systemic neglect of menstruation and the menstrual cycle”, “When “the private” becomes public: menstrual management” and “Navigating menstrual health: between medicalization and agency”.

### Systemic neglect of menstruation and the menstrual cycle

There were two sub-themes regarding the systemic neglect of menstruation and the menstrual cycle: “Being “othered”: the invisibilization of menstruation and the menstrual cycle”, and “Why am I bleeding from down there?”: learnings on menstruation and the menstrual cycle”.

#### Being “othered”: the invisibilization of menstruation and the menstrual cycle

Participants treated menstruation as a taboo topic. It was socially considered “dirty” and against “women’s purity”: *“It* (menstruation) *reminds you that it is an animal body, so then all this idea of an idyllic and virginal woman, so Virgin Mary does not exist (…), this stereotypical idea of a woman as all purity (…), so the period breaks that* (image), *because suddenly it is dirty and it bleeds and this is all opposite to the image of the clean and pure woman”-P27.* Some participants mentioned how they were expected to conceal menstruation, especially in public as it was “something intimate”. Participants often refer to physical discomfort or illness instead of disclosing menstrual pain or other menstrual-related issues, as a way to conceal menstruation. Interestingly, many participants mentioned that they did not perceive menstruation to be a taboo for themselves and the same time, words like: “menstruation” “vagina” or “vulva” were often omitted. The words used instead were “*period*”, “*down there”,* “*there*” or simply paused for a moment to ensure the interviewer understood what they meant. Talking about “women-things” in front of men was uncomfortable for some and it was mostly avoided. In general, menstruation was perceived shameful when they were younger and more acceptable to talk about during adulthood, although most women and PWM still feared staining in public: *“There was a girl in my school that had her period quite early and had very abundant periods and so she stained the chair with blood very often and all that (…) and so she told us to help her hide it, giving her a jacket, waiting for the boys to go to the playground so that we could later clean the chair before they saw it* (the chair) *like that”-P18.*

When discussing the first photograph, as part of the photo-elicitation, participants thought it was “brave” to menstruate in public. This act was seen as undefeated and empowering: *“I think it’s wonderful. And well, in fact, I see it as a vindictive act, right? (…) Very interesting, that she feels free. Of course, why would she stop running simply because her period has started”-P14.* At the same time, some participants assumed that menstruating in public could only be an accident, implying that no one could actively decide to display menstrual blood in public spaces. They expressed empathy and compassion for “this incident”, while they thought public displays of menstruation were socially seen as unhygienic and dirty; *“She is a pig, not wearing anything* (menstrual products)*”-P1.*

Some participants explained that menstrual taboo was related to women being historically seen and treated as an object for social reproduction (i.e., self-care and care for others, including maintaining physical spaces and organising required resources to care, and human reproduction). Blood had negative associations with death, pain, hurt and satanic rituals. A few participants mentioned that bodily fluids had different connotations depending on where they come from. Those that came from the vagina were stigmatized: *“It’s as if I’m sweaty and there’s a mark in my armpit, it is ok, but if it is a blood stain from the vagina, it’s not* (ok)*…”-P12.* Participants also mentioned the role of religion, politics, gender roles and patriarchy to contribute to the invisibilization of menstruation: *“As I said, religion (…) and I guess that they are things that have been perpetuated in society. Also, seeing women as something that has to be at home, really pulled away (…) it* (menstruation) *turns into something that has to be there, behind closed doors”-P19.* Furthermore, one participant, who identified as non-binary, explained how what is not considered “normative” in an androcentric society is relegated to the private sphere, mentioning the invisibilization of people who menstruate who identify as men: *“The androcentric society is sexist,… it is created based on androcentrism around the idea of the cis man, normative body, and everything else is relegated to the private sphere and also (…) bodies that can menstruate, for instance, (…) men who menstruate are totally invisibilised, and for example if they gestate they are directly seen as monsters and aberrant”-P18.*

Participants also indirectly referred to the stigmatization of “the menstruating woman”, seen as hysterical and irrational beings unable “to control themselves”: *“Menstruating woman equals hysteria and hormonal lack of control. (…) there might be a hormonal dysregulation (…) but that doesn’t mean that I do not know how to control myself (…) I think this is all a structural problem”-P23.* In line with this, a few participants referred to their partners as more reliable sources than themselves, to determine whether or not they experienced emotional changes throughout the menstrual cycle: “No, I don’t experience (emotional) changes, this is something that my husband can answer, he will say yes. No, no, I don’t experience them… (…) No, because look, I’ve just had my period and he hasn’t said anything to me… right now… he hasn’t said anything to me. No, my period just ended and he hasn’t said anything, so no, no… I don’t think I experience changes.”-P33.

#### “Why am I bleeding from down there?”: learnings on menstruation and the menstrual cycle

Menstrual education were generally depicted to be insufficient and often late. Participants learned about menstruation mainly through informal education (family, friends, and others). Even if participants appeared to have had access to positive learnings on menstruation, they described how they still held negative perspectives on menstruation when they were young: *“I still don’t understand very well… I remember that my mum had not had many period problems and so… that’s why I’m telling you that I don’t know where this idea that “periods are shit” came from, you know?”-P21.* Most participants cited their family members as their first and most accessible source for menstrual education: *“I know more or less what it is, menstruation, because in my house…. My grandmother had talked about it… or my mother had explained it to me. If I had to ask, I would ask and they would explain it to me”-P26*. Menstruation was almost exclusively discussed with other women and PWM.

Not all participants had access to formal menstrual education. The formal education encountered was mainly at school, where the focus was on reproductive and sexual health more than menstrual health. Participants mentioned that boys were rarely included or participated in menstrual education, which was criticized by some who demanded menstrual education regardless of sex/gender. Menstrual education should be adapted to each person and include all options for menstrual management (including free bleeding). Although most participants did not consider school an important source of information, it was for one participant: *“Luckily at school they already tell you about these things, so I was not caught off guard* (at menarche)*”-P11.*

Interestingly, most participants considered having learned more about menstruation and the menstrual cycle through their own experiences and being autodidactic: *“Like everything (…) One learns to walk by walking… so… every month I learnt something, mainly to understand your body (…) But I’m not conscious of it until… relatively until recently”-P33.* Social media was used as a platform for menstrual education during adulthood by some participants. However, access to menstrual education was mentioned to be unequal, as it was dependent on one’s family, the school they attended, and their access to self-learning. Some women explained how it was more difficult for them to access to menstrual education pre- and post-menarche in their countries of birth.

Some participants did not know what menstruation was at menarche. Some learned about menstruation over time through friends or other sources (e.g., the internet or TV): *“Yes, the period (…), they have not explained it well (…). Until I searched for “why is blood coming out from me down there”, nobody ever explained this to me”-P30.* Menarche was symbolically perceived as a transition into womanhood and adulthood. This was often perceived to be distressing (and even traumatic), especially if menarche was at an early age and they were the first ones in their friend groups to menstruate.

Participants generally had no information on how a healthy menstruation and menstrual cycle looked like, so participants appeared to construct their perceptions and believes comparing their experiences to women and PWM around them:*” I don’t have the ideal menstrual cycle that everyone dreams of, but I have also seen other realities that are worse”-P11.* If participants had questions or concerns, they usually turned to friends, family, and the internet before asking a healthcare professional.

Overall, participants agreed that menstrual education should be fully available and accessible, to promote positive views on menstruation and the menstrual cycle; men should be included. Those participants who were mothers reflected on how they would educate their children on menstruation: *“Well me with my child* (male child)*, I want to speak with him mainly like this, an open conversation and all and explain things not to repeat mistakes…* (referring to her parents)*”-P34.*

### When “the private” becomes public: menstrual management

Three sub-themes were identified, related to menstrual management and its impact on participants’ lives: “Managing menstruation in public”, “Menstrual products are necessity goods: menstrual poverty”, and “Menstruation’s impact on daily life: social participation and paid labour”.

#### Managing menstruation in public

Overall, menstrual management was perceived as “uncomfortable” and “annoying”, and to create a mental load women and PWM had to endure. In general, participants shared that managing menstruation at home was easier: *“I know that I am comfortable at home (…), I change it* (menstrual product) *much more often, but if I am at work instead, I know that I cannot get up every now and then to go to the bathroom and such”-P29.* In fact, managing menstruation in public spaces was deemed difficult by participants, mostly due to the lack of adequate facilities. Consequently, managing menstruation in public was generally seen as unhygienic, associated with health problems (especially if menstrual products could not be changed timely and hygienically), and avoided by some. In order to be considered adequate, menstrual management spaces had to be clean, contain a sink, a bin and a hanger for clothes within the bathroom stall, and have a door that could be properly locked.

These struggles were also common in workplaces and schools, not just because of the lack of appropriate menstrual management facilities but social barriers. P26 shared how she was questioned about her reasons for using the bathroom, a basic need, in school: *“So you had to tell them, please, I need to go to the bathroom because I’m on my period, and I need to go to change well, it’s like… you have to explain everything that happens so that they let you go… and of course you had to say it in the middle of the whole class, like they were like “wow, she has her period, she's going to change” … it was like … pf … you know, some uncomfortable moments”-P26.*

#### Menstrual products are necessity goods: menstrual poverty

All participants considered menstrual products as necessity goods, although one participant (P9) mentioned how they were only perceived to be necessary since menstruating in public was socially unacceptable. Most considered menstrual products unjustly expensive. They thought that taxes on menstrual products, and/or their price, should be lowered: *“They have super high taxes, when it is a basic need’s product”-*P24. While some participants advocated for the need of free menstrual products, at least for more vulnerable people, a few claimed that menstrual products should not be free as other essential products are not. Two participants (P33, P34) expressed their concerns if menstrual products were free; they said that this measure would need to be implemented carefully, implying that some people might re-sell menstrual products: *“That is a complicated topic because everything that is for free I believe that in some way people try to take advantage (…) There would need to be a lot of control. Too much control to start giving out things for free and that they do not escape to the “black market””-P34.*

Some participants had experienced menstrual poverty (P1, P3, P9, P18, P19, P25, P26, P29, P30, P31) at some point in their lives. P1 shared that she had struggled to afford menstrual products sometimes if menstruation came before her salary, or if the usual cheap pads were sold out. In these cases, she would use toilet paper or borrow menstrual products from friends. Menstrual products’ price seemed to be the main reason for participants not being able to buy the products they preferred, even if they caused discomfort and health issues*: I do not buy the pads non-branded. But I do buy the tampons non-branded. Maybe that's why it hurts* (laughing*) (…). Tampons* (branded) *are more expensive than pads* (branded)*. Buying the two things* (branded) *is not feasible for me to be honest.”-P30.* Other participants used menstrual products for a longer time than recommended when they could not afford menstrual products, or to save money. When having more financial issues, other participants decided to use the cheapest products available. However, a few participants attributed using cheap products to vaginal infections. Still, a few participants chose the cheapest products that did not cause them health issues.

On the other hand, a few participants had been forced to prioritize purchasing menstrual products over other goods or activities. Menstrual products were prioritized as they were considered necessity goods: *“So I knew that, (…) I am a woman and that it* (menstruation) *came every month. So, I knew that food and my* (menstrual) *product could not be missing. Then I would see about the rest”-P29.* Another participant shared a similar story: *“It was either pads or going for a bite? The pad goes first”-P30.*

Accessibility to menstrual products was also mentioned to be compromised depending on the type of product used and whether women and PWM lived in rural or urban areas. For instance, reusable, organic (without endocrine disruptors) and non-reusable products were more difficult to access, especially in small shops and rural areas. Assuming that women and PWM could always freely choose menstrual products was actually challenged by P14. She critically argued that the energy and time to look for less available products compromised the use of less mainstream (but healthier) options: *“It is not so much of a choice because I…, if all products were on the shelfs (…) And you really have them equally accessible, you are deciding more freely indeed. But if they are offering you a particular product that has a way higher cost. Well you have to find the time to do these things* (find menstrual products), *but in the end you don’t have it* (time)*, to be honest. And you go and look for the quick solution”-P14.*

Migrant and second-generation migrant participants also described how it was harder to access menstrual products in their countries of origin (Morocco, Pakistan, Jordan, Brazil and Philippines), especially tampons, organic and reusable products (except for cotton cloths or self-made menstrual pads). This was negatively linked to menstrual health: *“Unfortunately there are many women in other parts of the world, we are not talking only in Europe where we have everything in our reach. But in other areas (…) then, of course, they can’t feel the menstrual health”-P25.* The idea that reusable products have to be promoted was shared by some participants, although personal choice needed to be respected and free-will ensured. Others mentioned the importance of men being involved in promoting menstrual products’ accessibility.

#### Menstruation’s impact on daily life: social participation and paid labour

Menstrual pain appeared to have the biggest impact on participants’ lives, but also heavy bleeding, low energy levels, difficulty concentrating, premenstrual symptoms and emotional changes throughout the menstrual cycle. Even if participants mostly referred to the impact of menstruation on paid work, social participation was also mentioned. The latter were mostly adapted, but sometimes cancelled. Avoiding hard physical activities, sex, swimming, praying, going to the beach, exercising, and driving due to pain and/or heavy bleeding was common. Feeling unwell during menstruation or at any other time during the menstrual cycle had also an impact on other responsibilities such as those related to social reproduction: *“Obviously our health is dynamic and our emotional state. And our responsibilities outside of the workplace”-P10.*

Especially menstrual pain, but also emotional fluctuations related to the menstrual cycle and premenstrual symptoms, had an impact on paid labour. Many said it was more complicated to work while menstruating, due to the unavailability of spaces to change menstrual products and because of menstrual pain impacting their ability to focus. Presenteeism was common as absenteeism due to menstruation and was perceived as embarrassing and even unacceptable. This was due feeling that menstruation was not a “good enough reason” to stop working. In order to avoid absenteeism, participants either took painkillers (*“When I feel the slightest bit of pain, I take something* (painkillers), *because if not, I already know that I will not be able to work, you know? (…) I know I should not do it like this. But if I don’t do it… Every month I would miss two days of work, you know”?-P12)* or used HC to continue being productive at work, or to avoid managing menstruation in the workplace (*“I cheat, because my period comes on Friday night, (…) because at the beginning I did have a little bit of a painful menstruation and I believe that it came in the middle of the week (…) and work-wise (…) that day I was useless”-P6*)*.* However, some mentioned having to take days off from work. For all, there was more presenteeism at work, while during their time at educational institutions there was more educational absenteeism. They had also experienced presenteeism at school/university when responsibilities (e.g., an exam) were unavoidable. One participant (P25) gave her daughter painkillers for menstrual pain to prevent her daughters’ school absenteeism. She explained that if her daughter could not attend school due to menstrual pain, she would not go to work.

As P8 explained, menstruation is wrongly perceived to be a burden as it does not fit into the current socio-economic system based on continuous productivity. She suggests the need of systemic changes towards a more cyclic productivity model based on the menstrual cycle: *“Because you are as if you were ill, and ill people do not produce, and those who do not produce are less valued in society, and consequently in the working area it* (menstruation) *is seen as a burden (…) there is much that can* (menstrual cycle) *can contribute to qualitatively”-P8.* One woman (P34) told the researchers how some work colleagues had been fired due to menstruation supposedly impacting their work performance. Most participants stated for the need for menstrual policies at workplaces. Working from home during menstruation was seemingly perceived ideal by most participants. Menstrual leave was mentioned too, for those who may need it *“without having to feel bad, guilty, or that I’m a bad worker, or lazy”-P14.*

When asked if they thought menstruating created a disadvantage compared to men and non-menstruating people, some participants perceived it as uncomfortable more than a disadvantage. Other women and PWM thought menstruating was a disadvantage if they experienced dysmenorrhea, because of the mental load of managing pain, or if their partners did not support them. Others said that society had created the disadvantage, rather than it being originated from menstruating itself: *“Maybe we have to take a step back and see that the problem is not menstruation. Maybe it’s what we do with it…”-P12.* The lack of research was also mentioned by one participant as contributing to the invisibilisation, lack of knowledge on menstruation and the menstrual cycle and subsequently resulting in a perceived disadvantage of menstruating.

### Navigating menstrual health: between medicalization and agency

This theme includes data on participants’ accounts of two these two sub-themes: “Experiences and access to health services for menstrual health” and “Systemic medicalization of menstruation and the menstrual cycle”.

#### Experiences and access to health services for menstrual health

Overall, participants had previously sought medical help for menstrual-related issues, although some sought professional assistance only if they had issues getting pregnant. Despite a few participants mentioning that they had very good experiences accessing healthcare services, most shared negative experiences. They felt that they were not listened to and their concerns were easily disregarded and unattended. For instance, menstrual pain was normalized and dismissed within the healthcare system (as it was socially). Besides, health professionals often did not give enough information for women and PWM to make informed decisions: *“Gynecologists, and my mum is one of them, do not always explain well how hormonal contraception works in the body, right? And the truth is that it is important that this is explained, and once you know how it works, you decide”-P21.*

A participant, who was born in Pakistan and worked as a cultural mediator in healthcare services, mentioned that disclosing vaginal pain or amenorrhea was difficult for some Pakistani women to share with healthcare professionals (P31). This was aggravated if the healthcare professional was a man. Another participant stated that not all women and PWM have the same access to healthcare services, and thus to have a good menstrual health: *“I’ve you’ve got money you can afford it (…) economic differences can lead to differences in health, so, you can care for yourself more or less (…) You can attend to a doctor because you have less worries in relation to obtaining food or proper housing and so, and at the same time you can buy better-quality products or that better adapt to your menstruation”-P23.*

Participants reported to have been diagnosed with several menstrual-related issues or health conditions: endometriosis, polycystic ovary syndrome, amenorrhea, adenomyosis, dyspareunia, anemia or acne. Many participants experienced dysmenorrhea and heavy bleeding. Menstrual pain, already mentioned as the most invasive menstrual issue in participants’ lives, was managed either by trying to relax and use natural methods for pain management (e.g. hot water bottles or teas), by taking painkillers or HC, or by disregarding it: *“It depends if I’m at home or I’m out of home doing things, obligations that I cannot say no to. If I can I always try to rest (…) at home I can allow myself to be lying down (…) I use a hot water bottle or maybe I drink teas or hot things and I try to manage pain mentally. When it’s not possible (…) I would need to take a pill, some sort of painkiller, to do my tasks, otherwise it is impossible for me”-P8.* Interestingly, both menstrual pain and abundant bleeding were often considered normal and “part of being a woman” for most participants.

The perceived lack of answers from the healthcare system appeared to leave participants to navigate MH on their own, while some had sought help from naturopaths and other non-medical professionals: *“In reality, if I can avoid it I prefer no to go to traditional doctors (…) I would try to look for something like Chinese medicine, acupuncture, things like that”-P18.* Managing stress and emotional distress, factors that a few participants associated with changes in menstrual patterns (e.g., intensified menstrual pain), was an approach for some participants. Making nutritional changes had also helped one participant (P8) to manage menstrual pain. For the same participant, having to manage pain on her own had led her to feel guilty for feeling pain at times.

#### Systemic medicalization of menstruation and the menstrual cycle

Participants generally expressed frustration over treatment options offered in healthcare services. Medicalizing with HC appeared to be the only option given: *“It’s like the only thing* (HC) *that they give, the alternative to everything that happens to you with your menstruation is the “antibabies”-P12.* HC was often prescribed as a “solution” for menstrual-related issues such as menstrual pain, irregular menstrual cycles, heavy bleeding, amenorrhea, and acne. Most participants distrusted the generalized prescribing of HC as an actual treatment for their health concerns, especially when they had no medical exams or tests to explore the reasons for menstrual-related issues. There was a general perception of HC being “harmful” and some participants expressed resistance to taking HC for reasons other than contraception: “*If I will have a terrible acne breakout again… Uhm, I’d be looking for other ways to treat it”*-P5. Still, many perceived HC as an “easy” and accessible option to manage MH. Many participants disclosed negative experiences with HC due to the secondary effects, which were often overlooked by healthcare professionals. P31 shared not being aware of the risks of taking HC until they told her to stop taking HC due to a blood clot: *“it was a small blood clot, right? (…) Nobody had ever explained this* (risk) *to me”-P31.*

At the same time, medicalization was sometimes perceived as a means to gain control over their menstrual cycle, or to have a “normal” menstrual cycle. Some participants perceived HC to “regulate” their menstrual cycle. P3, who used the vaginal ring, said the following: *“That caught my attention, “wow, my period* (cycle) *was very controlled”-P3.*

Similarly, there was resistance to taking painkillers for menstrual pain, although these were often considered indispensable to continue daily life. Seeking professional help for menstrual pain was sometimes perceived as difficult and frustrating: *“I think it shouldn’t be normal for a woman to have to suffer these pains for menstruation… but as the specialists I have seen, told me the same thing, it has been well, so… whatever, I guess I will have to accept it (…) It is as if they don’t give importance to your pain, and (…) it makes you a bit angry, right? “-P12.*

## Discussion

Through participants’ experiences, we have explored the complex, structural and multi-dimensional nature of MH and MI. Menstrual inequities are an unacceptable consequence of androcentrism [9, 10]. They appear to be widespread among women and PWM and to take a variety of forms. Our findings suggest that MI does not only compromise MH but it may also impact emotional health and wellbeing [11]. While it was not as evident in our data, MI may also be a barrier to self-care and social reproduction [57]. As one participant explained, menstruation and the menstrual cycle are expected to remain in the shadows, hidden and managed in intimate and therefore private spaces. The parallelism with women’s historical relegation to the “private life” becomes conspicuous. While menstruation remains invisible, we argue that menstrual management in public spaces (including schools and workplaces) is challenging [58]. In turn, the lack of adequate menstrual management facilities in public spaces contributes to the invisibilization and taboo of menstruation. Besides, not being able to manage menstruation reinforces and perpetuates MI and may lead to health risks [18, 59–61].

With menstruation being socially stigmatized [11, 53, 62] and menstruation socially-constructed and expected to be invisible and hidden, controlling menstruation, for example through medicalization, becomes a moral imperative [19]. The medicalization of women has already been discussed in the literature [24, 25, 38, 63, 64]. Unsurprisingly, menstruation has for a long time been constructed as a “health issue” in need of medicalization [64] and has been critically under-addressed in public health [23]. This points to the importance of reframing menstruation as a vital sign of health [1], which does not seem implemented in clinical practice based on our results. There is a need to address the medicalization, not only of menstruation but of the menstrual cycle. Also, it is important to de-normalize menstrual pain and other menstrual-health related issues, to ensure timely diagnoses and respectful approaches to menstruation and MH. Working on shifting power dynamics in healthcare [24, 27] is also necessary to ensure women’s and PWM’s agency to care for their MH and overcome MI. Overall, the dismissive and often paternalistic approach towards MH could result in self-medicating, as seen in our results, and have a profound impact on general physical, emotional and community health. Inequities among different populations (e.g., vulnerable migrants, refugees, people from the LGTBIQ+ and Roma communities, those living in a situation of homelessness or functional diversity groups) to access healthcare services should also be acknowledged and tackled.

Among our participants, menstrual education appear to be gained mostly through social networks and self-learning. Although social learnings can strengthen agency among women and PWM, they could also lead to misconceptions around the menstrual cycle and menstruation [21]. It thus appears necessary to promote menstrual education in formal, such as schools or healthcare services, and informal settings. Both formal and informal education could equip individuals and communities with enough informational resources, to ensure good-quality community learning and development [65]. Implementing menstrual education in schools equally will be key to prevent deepening in-group MI [66]. We further suggest focusing on improving body literacy [27, 37]. However, it is also critical that social, economic and political systems do not ignore their responsibility in ensuring good-quality education and healthcare [67]. Boys, men and people who do no menstruate should also be included in educational strategies, as they could actively support women’s and PWM’s MH and become reliable partners in fighting MI.

Based on our findings, we propose three main types of menstrual poverty: (1) not being able to afford menstrual products, (2) not being able to choose preferred products, and (3) having to prioritize menstrual products over other products or activities. While menstrual policies have been implemented in some countries, these policies solely focus on menstrual products price reduction and availability. These strategies come with the risk of being tokenistic and not necessarily ensuring affordability [33] or addressing other aspects of MI. Even if promoting reusable menstrual products can be a sustainable and convenient option to reduce menstrual poverty and support MH [68], these products are not acceptable for all [69]. Furthermore, our findings suggest that accessibility to products may not just be related to price but availability. Women and PWM in a situation of financial and/or social vulnerability, or those living in rural areas, may have decreased opportunities to access less conventional products due to a lack of resources, including time poverty [70]. In turn, time poverty may limit educational opportunities, engaging with healthcare services, the ability to earn money and is associated with poorer health outcomes [10]. Thus, lack of time can be a barrier for MH and sustain MI among those already more vulnerable to experience it.

School presenteeism and absenteeism may have a long-lasting impact on women’s and PWM’s educational attainment, social and economic participation, and ultimately health outcomes [71, 72]. Other studies have assessed productivity loss in the workplace due to menstrual-related issues [30]. However, it is important to shift the narrative from attempting for women and PWM to fit into androcentric socio-economic systems, towards adapting social and economic productivity systems to those who have a menstrual cycle and menstruate. Workplace menstrual policies, such as flexible hours, menstrual leave or the creation of well-being spaces, are suggested as measures to support menstrual management in the workplace [73]. While it is important to acknowledge some women and PWM prefer to work from home or take a day off while menstruating, menstrual leave comes with the risk of being a band-aid for a structural problems in healthcare access. Besides, workplace menstrual policies should be adapted to the characteristics of each occupation and individual context.

Further attention should also be placed on the importance of self-care and social reproduction when it comes to MH and MI, challenging the generalized prioritization of monetized powers and markets [74]. As discussed in “Results” section, presenteeism and absenteeism from educational and productivity systems may not only come from one’s own menstrual experiences but the experiences of those one cares for. This is one of the reasons why MI should concern us all, regardless of whether we menstruate or not. Increasing community awareness and engagement for MH and MI could help ensure (self-)care among women and PWM. It could also support the establishment of informal and community care networks to guarantee social reproduction. Not only economic but public and social participation (e.g., engaging in social activities) appears to be compromised due to MI and MH issues, so policies need to ensure women’s and PWM’ social, economic and political participation. Further research is needed, particularly on the potential relationship between MI, MH and social and community participation.

While policy interventions are necessary, these need to be multi-dimensional and aimed at structural determinants of MI and MH. Although ensuring accessibility to menstrual products and menstrual management in workplaces are necessary to reach menstrual equity, stand-alone strategies can be rather paternalistic and become a barrier for individual agency and social capital [73]. Together with ensuring equal access to menstrual products, strategies to address MI and MH should ensure community-based menstrual education and formal education on menstruation and the menstrual cycle. Biological and reproduction-based education should be accompanied by curricula centered in promoting positive views on menstruation, body literacy and women’s and PWM’s agency. They should strengthen healthcare professionals’ training and healthcare services to approach menstruation and the menstrual cycle as vital health signs. Community and population-level strategies would also need to be implemented to de-stigmatize and debunk taboos surrounding menstruation, at the same time that public spaces are progressively re-structured to allow for healthy menstrual management. Any strategies need to be inclusive to vulnerable populations and non-binary and trans people [75–77]. Resources for intersectional, gender-based and feminist research on MH and MI are also required to continue working towards reaching menstrual equity and ensuring MH.

## Limitations

There were a few limitations to the sample. Participants who were interviewed in ASSIRs may have felt restrained to share negative healthcare experiences. Also, while we intended to include participants with different educational backgrounds, many participants had completed university education. Another limitation is related to the inclusion of trans, intersex and non-binary people. Despite the team’s efforts to recruit a more diverse sample, only a few non-binary people participated. Functional diversity could have been considered during recruitment. Although actions were taken to lessen the impact of the digital divide, recruitment and data collection were mainly done online due to COVID-19 restrictions. This could have compromised participation.

## Conclusion

Our study suggests that MI is a multifaceted phenomenon that needs to be addressed as a public health issue, through research, to ensure social equity and MH. The link between MI and MH, that should be further explored. It is essential to develop multidimensional structural policies and interventions that focus on promoting women’s and PWM’s agency and community care networks. Community-based actions should be encouraged, to promote MH and reduce MI. Health professionals’ training is also imperative. Future research could focus on assessing MI among different populations and communities, using gender and intersectionality approaches. Research could also aim at developing, implementing and evaluating interventions and policy strategies. Research and actions addressing MH and MI need to be inclusive to non-binary and trans people who menstruate and vulnerable populations, and account for social determinants of health.

## Supplementary Information

**Additional file 1.** Critical Appraisal Skills Programme (CASP) criteria.

**Additional file 2.** Participants’ menstrual cycle and menstruation characteristics.

**Additional file 3.** Interview topic guide.

**Additional file 4.** Photographs used for the photo-elicitation interviews.

## Data Availability

The datasets generated and analysed during the current study are not publicly available to maintain participants’ anonymity and confidentiality, but are available from the corresponding author on reasonable request.

## Abbreviations

ASSIRs: Public Sexual and Reproductive Health Centres
HC: Hormonal contraception
MH: Menstrual health
MI: Menstrual inequity
PWM: People who menstruate
