# Supplementary material for: Experiences of menstrual inequity and menstrual health among women and people who menstruate in the Barcelona area (Spain): a qualitative study
Source: Reprod Health. 2022 Feb 19;19:45. doi: 10.1186/s12978-022-01354-5 (PMC8857732; doi:10.1186/s12978-022-01354-5)
Supplement: Supplementary file 1 — Additional file 1. Critical Appraisal Skills Programme (CASP) criteria. [file 12978_2022_1354_MOESM1_ESM.docx]

**Additional file 1**

Critical Appraisal Skills Programme (CASP) criteria (Long et al., 2020):

1. **Was there a clear statement of the aims of the research?**
   1. Yes, the study aimed at exploring menstrual health and experiences of menstrual inequity in WPWM aged 18-55 in the Barcelona Metropolitan Area.
2. **Is a qualitative methodology appropriate?**
   1. Yes, to understand experiences qualitative methodology is most appropriate.
3. **Was the research design appropriate to address the aims of the research?**
   1. Yes. The design and theoretical approaches taken to conduct the research have been appropriate and clearly identified in the article.
4. **Was the recruitment strategy appropriate to the aims of the research?**
   1. Taking the circumstances with COVID-19 into consideration, yes. However, since recruitment was mainly online, there is a risk of vulnerable populations being excluded. There could have been done more to include people of the trans* community.
5. **Was the data collected in a way that addressed the research issue?**
   1. Yes, through semi-individual interviews with photo-elicitation techniques. Semi-individual interviews were appropriate, and the use of photo-elicitation techniques can help facilitate a conversation that could be taboo for some participants.
6. **Has the relationship between researcher and participants been adequately considered?**
   1. Yes, authors applied reflexivity throughout the research so they have continuously reflected on how their own experiences, perceptions and privileges have affected the relationships with participants.
7. **Have ethical issues been taken into consideration?**
   1. Yes. Ethical approval was obtained from IDIAPJGol Ethical Committee on 21st Nov 2020, Ref 19/178-P.  Written and verbal consent were obtained prior to participation and audio recordings All participants were made aware of their participation being anonymous, confidential, and voluntary, along with their right to withdraw consent to participate at any given moment until data analysis.
8. **Was the data analysis sufficiently rigorous?**
   1. Yes. Data analysis has been triangulated, formulated, discussed with the research team, and discussed again and reformulated before reaching final data analysis. The analysis process has been described in detail to ensure transparency.
9. **Is there a clear statement of findings?**
   1. Yes, results are clearly stated and discussed. The conclusion along with the research, policy, and practice recommendations clearly explain the findings.
10. **How valuable is the research?**
    1. Considering that this, to the authors knowledge, is the first study on menstrual health and inequity in Spain it is very valuable. Menstrual inequity has been an invisible problem in Spain until this study, and menstrual health has been neglected.
