# Supplementary material for: Experiences of menstrual inequity and menstrual health among women and people who menstruate in the Barcelona area (Spain): a qualitative study
Source: Reprod Health. 2022 Feb 19;19:45. doi: 10.1186/s12978-022-01354-5 (PMC8857732; doi:10.1186/s12978-022-01354-5)
Supplement: Supplementary file 2 — Additional file 2. Participants’ menstrual cycle and menstruation characteristics. [file 12978_2022_1354_MOESM2_ESM.docx]

**Additional file 2**

Participants’ menstrual cycle and menstruation characteristics

|  | **Age at menarche** | **Perceived pain level** | **Cycle length** | **Perceived abundance** | **Factual abundance*** | **Menstruation length** | **Current use of hormonal contraception** | **Menstrual cycle and menstruation-related diagnoses** |
| --- | --- | --- | --- | --- | --- | --- | --- | --- |
| **P1** | 9 | High (varying) | 35-40 days | Very abundant | Unknown | Unknown | No |  |
| **P2** | 14 | High (varying) | Unknown (regular) | Very abundant | Abundant | 6-7 days | No |  |
| **P3** | 14 | Medium (varying) | Postpartum - prior, using HC, very regular | Abundant | Unknown | 7 days | No |  |
| **P4** | N/A | High | 30 days | Normal | Abundant | 5 days | No |  |
| **P5** | 13-14 | No pain | >45 days | Light | Light | 4-5 days | No | Possible PCOS** |
| **P6** | 14-15 | Pain when stressed | 28 days | Light | Light | 3-4 days | Yes (pill) |  |
| **P7** | 12 | High | 25 days | Abundant | Medium | 4 days | No |  |
| **P8** | 13 | High | 26-28 days | Medium | Medium | 3-4 days | No | Endometriosis |
| **P9** | 12-13 | High | 30 days | Abundant | N/A | 6-7 days | No |  |
| **P10** | 13 | Low/No pain | 30-31 days | Light | Light | 5 days | No |  |
| **P11** | 11 | High | 26-27 days | Normal | Medium | 5 days (3 days bleeding, 2 spotting) | No |  |
| **P12** | 9 | High | 28-40 days | Very abundant | Medium | Unknown | No |  |
| **P13** | 13 | Medium | 28-32 days | Normal | Medium | 6-7 days | No |  |
| **P14** | 13 | High | 28 days | Not sure | Abundant | 3-5 days | No |  |
| **P15** | 11 | High | 28-29 days | Light | Medium | 4-6 days | No |  |
| **P16** | 13 | High | Unknown | Very abundant | Unknown | Unknown | No |  |
| **P17** | 10 | High | 38-48 days | Very abundant | Abundant | 8-9 days | No | Dysmenorrhea; Long-COVID |
| **P18** | 12 | High | 35-40 days | Very abundant | Abundant | Unknown | No | Anemia; Long-COVID |
| **P19** | 11 | Medium | 28 days | Abundant | Medium | 5-6 days | No |  |
| **P20** | 12 | Very high | 29 days | Unknown | Unknown | 2-4 days | No | Endometriosis; Dysmenorrhea |
| **P21** | 11 | Medium | Unknown | Light | Medium | 3-4 days | No | Adenomiosis; Dyspareunia; Dysmenorrhea |
| **P22** | 12 | High | Unknown | Abundant | Abundant | Unknown | No |  |
| **P23** | 10 | Very high | 28 days | Very abundant | Abundant | 10 days | No | Endometriosis |
| **P24** | 14 | Medium (varying, sometimes high) | 32-42 days | Normal | Medium | 4 days | No |  |
| **P25** | 12 | N/A | 20 days-3 months | Abundant | Abundant | Unknown | Yes (implant) |  |
| **P26** | 11 | High | 35-42 days | Very abundant | Abundant | 3-5 days | Yes (pill) | PCOS, amenorrhea |
| **P27** | 13-14 | Low-medium | 28-30 days | Abundant | Abundant | 7 days | No | PCOS |
| **P28** | 14 | Low/No pain | 28-32 days | Abundant | Medium | 5-7 | No |  |
| **P29** | 13 | Low/No pain | 28-30 days | Normal | Medium | 5 days | No |  |
| **P30** | 14 | High (varying) | Unknown (regular) | Very abundant | Abundant | 4-7 days | No |  |
| **P31** | 12-13 | High | 25-27 days | Abundant | Medium | 5-9 days | No | Anemia |
| **P32** | 13 | High | 28 days | Medium | Medium | 5 days | No | Stroke due to hormonal contraception |
| **P33** | 13 | Low | Unknown | Very light | Light | 1 day | No | Blood clots |
| **P34** | 13-14 | Medium | 15-20 days | Very light | Light | 4 days | Yes (pill) | Endometriosis; Ovarian cyst |

* Abundant: ≥16 tampons or ≥ 20 regularly absorbed pads, or more than 4 filled 20ml menstrual cups in total each menstruation; Medium: 7-15 tampons or 7-19 regular absorption pads, or between 1 and 4 20ml menstrual cups filled in total each menstruation; Light: ≤ 6 tampons or pads of regular absorption, or less than 1 menstrual cup of 20ml filled in total each menstruation. **PCOS=polycystic ovary syndrome. [Based on: Munro, M.G., Critchley, H.O.D., Fraser, I.S., Haththotuwa, R., Kriplani, A., Bahamondes, L., et al. 2018. The two FIGO systems for normal and abnormal uterine bleeding symptoms and classification of causes of abnormal uterine bleeding in the reproductive years: 2018 revisions. Int. J. Gynecol. Obstet. 143, 393–408. <https://doi.org/10.1002/ijgo.12666>].
