# Supplementary material for: Experiences of menstrual inequity and menstrual health among women and people who menstruate in the Barcelona area (Spain): a qualitative study
Source: Reprod Health. 2022 Feb 19;19:45. doi: 10.1186/s12978-022-01354-5 (PMC8857732; doi:10.1186/s12978-022-01354-5)
Supplement: Supplementary file 3 — Additional file 3. Interview topic guide. [file 12978_2022_1354_MOESM3_ESM.docx]

**Additional file 3**

Interview topic guide

- Introduce researcher and institution
- Introduce research study (refer to participant information sheet)
- Remind about confidentiality and anonymity
- Ask for consent to audio-record the interview

| **Objectives** | **Questions** |
| --- | --- |
| Introduction | How would you describe yourself? |
| Explore the conceptualization of menstruation | What is menstruation for you?   - How do you feel about it? - What do you feel in relation to menstruation? - What are the negative aspects of it for you? And the positives?   What is menstrual health for you? |
| Explore experiences of menstrual health | How was it the first time that you menstruated?   - How did you feel? - How were the reactions of people around you? - Who did you speak with? - Have you felt that you could speak to your family about menstruation? - Did you feel ready to start menstruating?   Usually, how is your menstruation like?   - Menstrual pain (how is it? how do you manage it?) - Bleeding abundance (ask for quantity of menstrual products used in one menstruation) - Bleeding length each menstruation - Menstrual cycle length - Any related diagnoses (e.g., endometriosis) or menstrual-related issues - Use of menstrual products (what products do you use? why these products and no other?) - Use of hormonal contraception   Do you notice any physical and/or emotional changes throughout your menstrual cycle (so between one menstruation and the next)?   - If so, what changes do you experience? - How do you feel about these changes? |
| Explore experiences of menstrual inequity | *Menstrual education*  How have you learnt about menstruation?   - Do you feel that you have enough information? - How have you informed yourself since you started menstruating until now?   *Menstruation-related taboo, stigma and discrimination*  [Show photography 1, marathon runners]   - Could you describe this photograph? - What do you feel looking at it?   Do you think that menstruation is a taboo topic (a topic that people do not talk about or is ashamed of doing it)?   - If so, why do you think that is? - Does that happen to you?   *Impact on social and paid work*  How do you think menstruating may impact your daily life?  What activities do you avoid when menstruating (e.g., because of menstrual pain or being ashamed)?  Some people believe that menstruating may be a disadvantage (in relation to men/people who do not menstruate) at school or at work, what do you think about this?  Has it ever happened to you that you stop doing your day-to-day activities (e.g., not going to work or school) when menstruating? Can you tell us about it?  Some participants tell us that they feel the need to rest when they are menstruating and they are not as productive or have trouble concentrating, what do you think about this?  *Access to healthcare services*  Have you ever sought help from a healthcare professional for any menstrual-related questions or issues?   - How has your experience been? - Have you spoken to someone else about it? - Have you attended any other services/sought help from someone else?   *Economic barriers to access menstrual products*  What do you think about the price of menstrual products? |
|  | Some people have told us about having economic problems and not being able to afford menstrual products, has this ever happened to you?  Has it ever happened to you that you could not choose what menstrual products to use due to financial issues?  Has it ever happened to you that you could not afford other goods or services to be able to get menstrual products?   - If so, how have you felt about it?   How have you access menstrual products generally?   - Have you ever had problems finding the products that you would like to use? - Have you had issues accessing menstrual products during the COVID-19 pandemic?   Have you ever used menstrual products more time than recommended because you could not access another menstrual product?  Have you ever used menstrual products more time than recommended because you could not access facilities to change?  [Show photography 2, Mexican congresswomen]   - Could you describe this photograph? - What do you feel looking at it? |
| Explore perceived impact of COVID-19 and the COVID-19 pandemic on menstrual health | Have you noticed any changes in your menstrual cycle or menstruation since the start of the COVID-19 pandemic?   - If so, what changes have you experienced? - If so, how have you managed them? - If so, have you sought professional help? How has your experience been accessing healthcare services?   Have you had COVID-19 (diagnosed/suspected)?   - If so, have you experienced symptoms for over 4 weeks (long COVID)? - Have you noticed any changes in your menstrual cycle or menstruation since you have had COVID-19? |
| Identify opportunities to improve menstrual health and menstrual equity | What do you feel would need to change to improve menstrual health in the population?  What do you feel about reducing menstrual products’ taxes?   - And about reducing their price? - And about providing them for free?   How could the social disadvantages due to menstruating be reduced? |

- Request written consent (sign consent form)
- Would you like to add anything else?
- Would you like to ask us any questions?
- Thank them for participating
- Give out debrief form and voucher
- Explain projects’ next steps and dissemination of findings
