# Supplementary material for: Experiences of menstrual inequity and menstrual health among women and people who menstruate in the Barcelona area (Spain): a qualitative study
Source: Reprod Health. 2022 Feb 19;19:45. doi: 10.1186/s12978-022-01354-5 (PMC8857732; doi:10.1186/s12978-022-01354-5)
Supplement: Supplementary file 4 — Additional file 4. Photographs used for the photo-elicitation interviews. [file 12978_2022_1354_MOESM4_ESM.docx]

**Additional file 4**

Photographs used for the photo-elicitation interviews


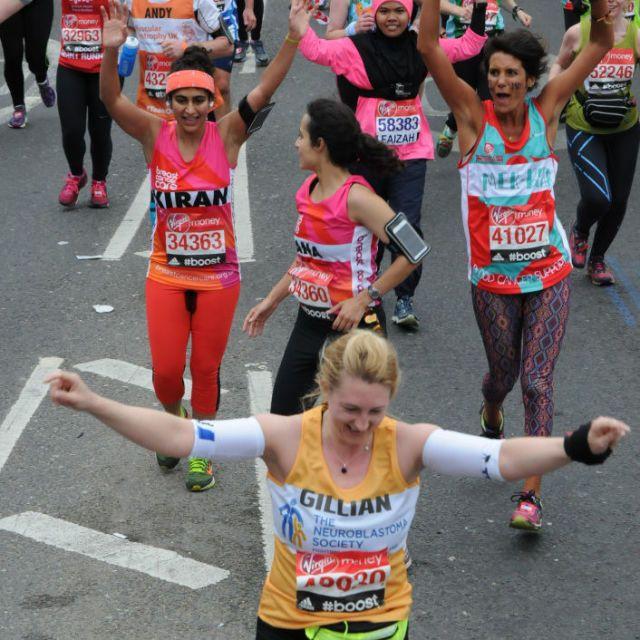


Figure S1. Marathon runners. Kiran Gandhi in the London Marathon with her friends. Photograph from: <https://madamegandhi.blog/2018/12/21/madame-gandhi-featured-in-allures-100-years-of-period-campaign/>


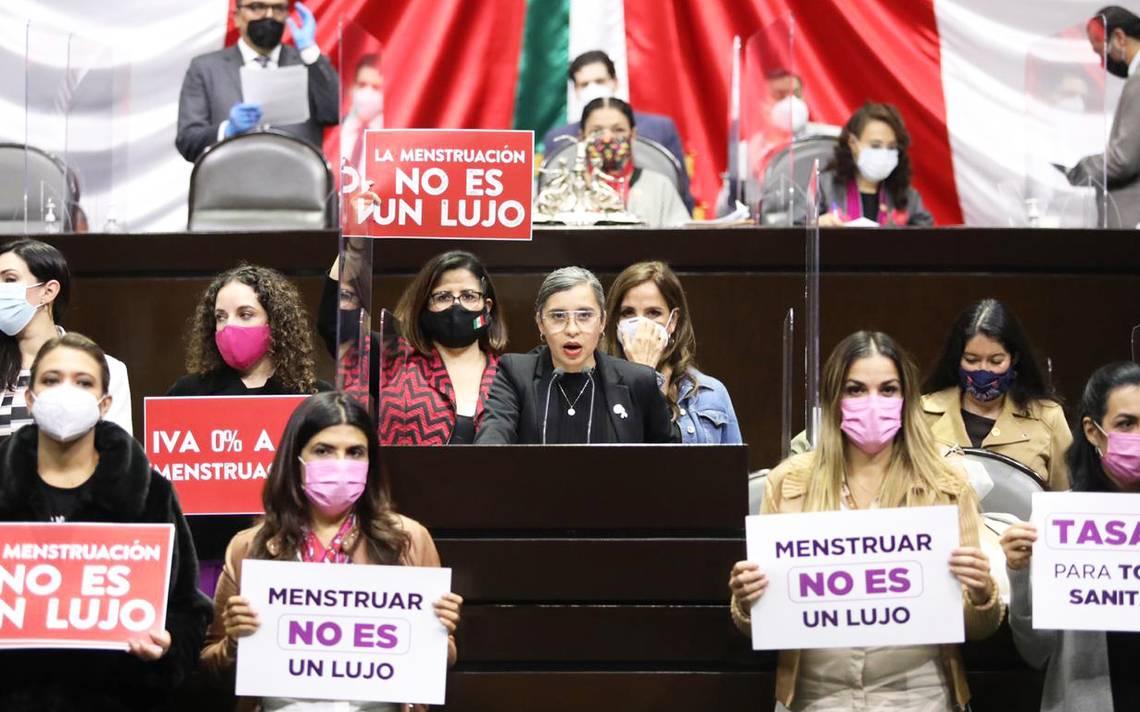


Figure S2. Mexican congresswomen and Menstruación Digna México (@dignamx) representatives. Image from: [*www.elcafediario.com*](http://www.elcafediario.com)
